# DEATH FEARS AND ANXIETY ACROSS THE LIFE COURSE: IMPLICATIONS FOR DEATH REFORM

**DOI:** 10.1093/geroni/igac059.1387

**Published:** 2022-12-20

**Authors:** Amber Thompson, Rebecca Utz

**Affiliations:** University of Utah, Sandy, Utah, United States; University of Utah, Salt Lake City, Utah, United States

## Abstract

Western culture is experiencing a movement for death reform which includes aspects such as death acceptance and supporting individual choice concerning how one wishes to experience end-of-life (EOL). The underlying goal of this reform is to foster natural curiosity about death and dying in order to soothe death fears and anxiety. Over the past few decades in the U.S. there has been an increase in public support for the EOL option of medical-aid-in-dying (MAID), or legally prescribed pharmaceuticals intended to cause death. There have been mixed findings regarding the effects of age on attitudes and perspectives towards death and dying. The aim of this study was to determine the relationship between age, death fears and anxiety, values and perspectives, demographic characteristics, and approval of MAID at EOL in a U.S. nationally representative sample (N = 1994), using a partially latent structural equation model (SEM). Results show that age has a curvilinear effect on death fears and anxiety, and that as death fears and anxiety increase support for MAID decrease. This suggests that the ability to provide person-and-family-centered care at EOL must address the biopsychosocial aspect of death fears and anxiety across the life course. These findings demonstrate the need for more research documenting changes in attitude and perspectives towards EOL for different populations (i.e., ages). The potential benefits of this approach provide a basis for expanding education and awareness of both death and dying in general, and EOL needs and wants for older adult populations.

